# Exercise-Induced Muscle Damage and Running Economy in Humans

**DOI:** 10.1155/2013/189149

**Published:** 2013-02-04

**Authors:** Cláudio de Oliveira Assumpção, Leonardo Coelho Rabello Lima, Felipe Bruno Dias Oliveira, Camila Coelho Greco, Benedito Sérgio Denadai

**Affiliations:** Human Performance Laboratory, UNESP, Avenue 24 A, Bela Vista-Rio, 13506-900 Rio Claro, SP, Brazil

## Abstract

Running economy (RE), defined as the energy demand for a given velocity of submaximal running, has been identified as a critical factor of overall distance running performance. Plyometric and resistance trainings, performed during a relatively short period of time (~15–30 days), have been successfully used to improve RE in trained athletes. However, these exercise types, particularly when they are unaccustomed activities for the individuals, may cause delayed onset muscle soreness, swelling, and reduced muscle strength. Some studies have demonstrated that exercise-induced muscle damage has a negative impact on endurance running performance. Specifically, the muscular damage induced by an acute bout of downhill running has been shown to reduce RE during subsequent moderate and high-intensity exercise (>65% VO_2_max). However, strength exercise (i.e., jumps, isoinertial and isokinetic eccentric exercises) seems to impair RE only for subsequent high-intensity exercise (~90% VO_2_max). Finally, a single session of resistance exercise or downhill running (i.e., repeated bout effect) attenuates changes in indirect markers of muscle damage and blunts changes in RE.

## 1. Introduction

 Running economy (RE), defined as the energy demand for a given velocity of submaximal running, is an important predictor of aerobic running performance, particularly in elite runners who have a similar aerobic power (i.e., maximal oxygen uptake, VO_2_max) [1]. Runners with high RE demonstrate lower energetic cost at submaximal velocity and consequently tend to run faster at given distance or longer at a constant velocity.

A number of biomechanical (e.g., gait patterns, kinematics, and the kinetics ofrunning) and physiological factors (e.g., oxidative muscle capacity) seem to influence RE in trained athletes [2, 3]. Moreover, some interventions (plyometric, resistance and altitude training) performed during relatively short periods of time (~15–30 days) have been successfully used to improve RE [4–6]. Plyometric and resistance trainings lead to neuromuscular adaptations such as increased neural drive to the muscles and changes in muscle stiffness and muscle fiber composition, which might reduce the energetic cost during submaximal exercise. However, plyometric and resistance trainings, especially when they are unaccustomed activities, may cause delayed onset muscle soreness (DOMS), swelling, and reduced muscle strength. The negative effect of muscle-damaging exercises on endurance running performance has been experimentally demonstrated in both animal [7, 8] and human experiments [9]. However, studies that have investigated the effect of exercise-induced muscle damage (EIMD) on RE have produced equivocal results [9–12]. This review discusses the effects of EIMD induced by different exercise types (strength, long-distance running, and downhill running) on RE. Different recovery strategies aiming to enhance the RE after EIMD are also addressed. 

## 2. Running Economy

Aerobic fitness, as well as running performance, can be measured by different variables, for example, maximal oxygen uptake (VO_2_max), lactate threshold (LT), onset of blood lactate accumulation (OBLA), movement economy (ME), or running economy (RE). The VO_2_max, which reflects an individual's maximal rate of aerobic energy expenditure, has been considered the gold standard for measuring aerobic power [13]. Indeed, the VO_2_max has a positive association with aerobic running performance obtained during middle- and long-distance events (1,500 m–42,195 m) [14–16]. However, some studies [17–20] have shown that subjects with similar VO_2_max values may attain different aerobic running performance or VO_2_ values during exercise of similar duration and intensity. These differences are most likely due to variations in ME among subjects.

The ME is defined as the amount of energy necessary (Kcal·min^−1^) to perform a given task [21]. However, due to the difficulty to determine the external work performed during running, RE, expressed as the volume of oxygen uptake (mL·Kg^−1^·min^−1^) during a specific submaximal running intensity (Km·h^−1^), has been adopted. There is a strong association between RE and aerobic running performance, with RE being a better predictor of performance than VO_2_max, particularly in athletes who have similar VO_2_max [19, 22].

Several factors have been proposed to influence RE in trained subjects. These include oxidative muscle capacity and muscle stiffness. Muscle stiffness corresponds to the ability of the muscles to store and release elastic energy. Moreover, some interventions such as training, environment, and muscle damage can modify the oxygen cost over a range of running speeds [6, 11, 23–25].

Improved oxidative muscle capacity can be associated with reduced oxygen consumption per mitochondrial respiratory chain during submaximal exercise. Trained subjects are known to have better RE than untrained individuals, and long-distance runners are more economical than middle-distance runners [26]. Additionally, a high weekly volume of training has also been associated with better RE. However, a short period (4–6 weeks) of high-intensity aerobic training (near or above VO_2_max) can also lead to improvement in the RE of trained runners [27]. 

In addition to aerobic characteristics and adaptations, neuromuscular profile factors have also been considered important aspects of RE. Type II muscle fibres seem to be positively correlated with submaximal energy consumption, especially at lower speeds [28]. Furthermore, both muscular stiffness and the ability to rapidly develop muscular force (i.e., rate of force development (RFD)) have demonstrated significant correlations with RE [29, 30]. Stiffer muscle-tendon complexes may increase elastic energy storage by reducing the ground contact time, thus decreasing the running oxygen cost. Similar to stiffness, a higher RFD is associated with a shorter time to generate a contraction. This effect could also diminish the ground contact time and running oxygen cost. Heavy weight and plyometric training associated with endurance training have improved RE in well-trained runners [5, 31]. Basically, these types of strength training induce neuromuscular adaptations such as increased neural drive to the muscles, altered muscle-tendon complex stiffness, and changed muscle fibre composition (i.e., I → IIA *←* IIX).

Environmental variables can also be used to reduce the energetic cost of running. RE can be improved (2-3%) after relative short periods (~15–20 days) of altitude exposure (~2.000–4.500 m). Altitude exposure during daily activities, sleeping, or training can enhance RE at sea level altitude through haematological and muscle changes in favour of oxygen transport [32–34]. Moreover, heat exposure during training sessions can also improve RE by enhancing the thermoregulatory process, thus reducing the cardiovascular and muscle work for a given exercise intensity [23, 35, 36]. 

More recently, EIMD has also proposed to generate important modifications in RE. Muscular damage induced by an acute bout of downhill running has shown to reduce RE in the days following the intervention (24–120 hours) [9, 11]. Specific aspects of this intervention are addressed hereafter. 

## 3. Muscle Damage and the Repeated Bout Effect

 Skeletal muscle damage has been considered an important factor contributing to DOMS and strength loss after eccentric exercise [37]. Basically, the exercise conditions at which muscle damage can be induced are unaccustomed exercises and exercises with higher intensity or longer duration than those to which the subject is adapted [37, 38]. The resulting metabolic overload and mechanical strain have been suggested the main factors generating muscle damage [38]. Warren et al. [39] have suggested that measures of muscle function such as strength and power are effective indicators of both the magnitude and time course of muscle damage. Depending on the magnitude of muscle damage, muscle force at isometric, and dynamic testing conditions may be impaired for 1–7 days after the exercise [40–43]. Other important symptoms of muscle damage are disruption of the sarcolemma and extracellular matrix [44, 45], increased blood levels of creatine kinase (CK) and myoglobin (MB), stiffness, and swelling [46–48].

 In general, muscle damage can be induced by both static (isometric) and dynamic (concentric and eccentric) muscle contractions. However, there is substantial evidence that eccentric muscle actions result in greater muscle damage than isometric or concentric actions [49–52]. The magnitude of strength loss after EIMD may vary between 5–10% and ~60% [43, 52], depending on the characteristics of the protocol and the type of muscle actions (i.e., isometric, concentric or eccentric) used during the posttest. The different effects of eccentric versus isometric or concentric actions have also been verified in the context of whole body exercises (i.e., running, cycling, and cross-country skiing) [43, 53]. For example, muscle damage and strength loss are higher during running (~20–30%), which involves concentric and eccentric actions, when compared with cycling (~10–15%), which involves mainly concentric actions [53]. In accordance with Millet and Lepers [53], although concentric and eccentric actions are present during cross-country skiing, muscular damage is considerably lesser during this exercise than in running because shock waves are present only during running. The main factors attributed to the greater effect of eccentric contractions on muscle damage are the higher peak torque values [54] and reduced motor unit activation for a given force [54–56], both of which induce a higher mechanical stress on the muscles [54]. Other important aspects of the greater muscle damage induced by eccentric muscle actions are that no energy (ATP) is necessary to detach the cross-bridges formed during muscle contraction [57] and that the longer length of the muscles during the contraction generates greater muscle damage. 

 In addition to the main mechanical factors (i.e., the force level produced and the change in muscle length) [37, 58, 59], some metabolic factors such as substrate depletion, calcium influx, and reactive oxygen species have also been proposed to influence muscle damage [38, 60]. The effects of the different mechanical and metabolic factors that would contribute to muscle damage do not occur at the same time. The time course of the events involves damage in components of excitation-contraction system and sarcomeres [59] and degeneration and regeneration of muscle fibres, during which DOMS, stiffness, and swelling occur [37]. Additionally, there is an inflammatory response generating a transfer of fluid and cells to remove damaged contractile proteins and cellular debris from the damaged muscles [61]. Thereafter, the muscle regeneration process is initiated [61]. Although some of these effects may appear only some hours after the exercise, muscle strength may be impaired during and immediately after the exercise. Thus, mechanisms other than muscle damage can also explain the muscle fatigue (i.e., strength loss). 

 It has been suggested that the magnitude of muscle damage and the loss of muscle function might be attenuated after one bout of eccentric exercise [62–64]. This concept is known as the repeated bout effect (RBE). The RBE has been demonstrated after both eccentric muscle actions [65] and downhill running [66]. In general, this protective effect is confirmed by the reduced decrements and faster recovery of muscle strength, less swelling and DOMS, and attenuated changes in CK and MB in the blood [62, 67, 68]. In addition, alterations in muscle circumference or echo intensity (inflammation) are also smaller after the first eccentric exercise bout [68]. This protective effect has been demonstrated after a few days of the eccentric exercise [66] and may last up to 6 months (circumference, DOMS, and inflammation) or 9 months (maximal isometric force, CK), depending on the marker of muscle damage [65]. 

 It has been hypothesized that the RBE is mediated by neural, cellular, and mechanical mechanisms [63, 64]. The neural changes proposed to contribute to the RBE are increased slow-twitch fibre recruitment and synchronisation of motor unit firing, better distribution of the workload among muscle fibres, higher participation of synergist muscles to torque production, and increased motor unit activity relative to torque produced [69–71]. Neural mechanisms have been suggested based on the reduced median frequency [69], which reflects some central aspects related to motor unit recruitment. Howatson et al. [69] have demonstrated a 10% decrease in median frequency 14 days after a bout consisting of either 10 or 45 maximal eccentric actions. RBE has also been observed in the untrained contralateral limb, referred to as the contralateral RBE [72]. These studies [69, 72] confirm that, in addition to intramuscular adaptations, central aspects regarding motor unit recruitment are also involved in RBE.

 The main mechanical adaptations associated with RBE are increased muscle stiffness and intramuscular connective tissue and changes in the intermediate filament system (maintenance of structural integrity of sarcomeres) [63]. Cellular adaptations are associated with higher number of sarcomeres in myofibrils [59, 73], which might decrease myofibrillar disruption in the next exercise bout, strengthened plasma membranes, increased protein synthesis, removal of stress-susceptible fibres [59, 74, 75], and remodelling of the cytoskeleton, including effects on proteins such as titin and desmin, talin and vinculin [76], which might improve the strength and the stability of sarcomeres and protect muscle fibers against injuries. Other adaptation that has been hypothesized to explain the RBE is the lesser inflammatory response. Since the mechanical disruption is decreased after the first eccentric exercise bout, the stimulus for the inflammatory response is also reduced after the exercise [73, 74]. Some of these alterations have been associated with reduced muscle damage (strengthened extracellular matrix) and a change in the optimal angle for torque production toward a longer muscle length (increases in number of sarcomeres). 

 The magnitude of muscle damage induced by eccentric exercise is greater at longer muscle lengths [65, 77]. When the muscles are elongated, the sarcomere length is also greater. Because the severity of muscle damage is influenced by the muscle strain generated [73, 78], it has been suggested that the RBE would be greater under conditions of longer muscle lengths. Nosaka et al. [73] have investigated the effect of the range of motion of the exercise used to induce muscle damage on the RBE. The protocol used to induce muscle damage involved 24 maximal eccentric contractions of the elbow joint, using amplitudes of 50–100° or 130–180°. Although the changes (maximal isometric strength, range of motion, upper arm circumference, muscle soreness, and CK) induced by the first bout were significantly greater using the higher amplitude, both exercise conditions induced RBE. However, the effect generated by the short range of motion was lesser than that promoted by the higher amplitude. 

 Other factor that can modify this protective effect (i.e., RBE) of eccentric exercise is the magnitude of muscle damage [65, 66], which is influenced by the exercise intensity of the first bout. Chen et al. [66] showed that 30 eccentric contractions performed at 40% of maximal isometric strength generated a smaller attenuation of the changes in indirect markers of muscle damage (20–60%) than maximal eccentric exercise (65–100%). 

 It has been also demonstrated that both the muscle damage level (i.e., CK) and strength impairment and recovery (i.e., isometric torque) are progressively greater with increases in the number of bouts (1–4). However, Chen et al. [68] have demonstrated that repetitive submaximal eccentric exercise bouts (40% MVC) performed every two weeks promote a protective effect similar to that induced by one maximal eccentric exercise bout. In this study, the main indirect markers of muscle damage were less affected by the second to the fourth bouts of submaximal eccentric exercise than the first; that is, the protective effect is promoted under conditions of reduced levels of induced muscle damage. Even after repeated submaximal bouts the magnitude of muscle damage was still smaller than that induced by one maximal bout. The authors suggested that the effect of exercise intensity on the protective effect of the first bout does not apply when some bouts of low-intensity exercise are performed. Therefore, the magnitude of muscle damage does not necessarily affect the protective effect of eccentric exercise. Moreover, Howatson et al. [69] have compared two protocols of maximal eccentric contractions to induce muscle damage with 45 or 10 contractions. After 14 days, subjects performed the same protocol with 45 contractions. Although the effect of the higher volume of the first bout on damage markers (CK, DOMS, and isometric torque) was greater, the protective effects of both protocols were similar. Therefore, the intensity of the first bout seems to be the main aspect of the magnitude of muscle damage and RBE. 

 Because one exercise bout is enough to generate the RBE, some studies have also investigated whether resistance training could also reduce the effects of eccentric exercise on muscle damage markers [67, 79]. Specifically, Newton et al. [67] found that resistance-trained subjects demonstrated smaller RBE when compared with untrained subjects. Moreover, Falvo et al. [79] did not find changes in indirect markers of muscle damage (maximal isometric torque and CK) in resistance-trained men. The authors attributed the absence of RBE to a lack of neural adaptation. Thus, it is likely that strength training induces to adaptations that reduce the RBE. 

 The majority of studies that have analysed the RBE used relatively short time periods after the eccentric exercise (i.e., from approximately 7–14 days to 6–9 weeks). However, some studies [80, 81] have reported that the RBE induced by 24 maximal eccentric actions of the elbow flexors may last up to 6 months. Nosaka et al. [80] aimed to investigate the responses of the main indirect markers of muscle damage (CK, maximal isometric torque, DOMS, and swelling) five days after the eccentric exercise bout, with sessions six, nine, and twelve months apart. The main finding of this study was that the RBE for strength, swelling, DOMS, and CK lasted up to six months. 

## 4. Strength Exercise, Muscle Damage, and Running Economy

 A variety of studies have investigated the influence of EIMD and DOMS on neuromuscular performance indicators (i.e., strength and rate of force development) [82–84]. These studies verified that the isomeric peak torque is compromised immediately after the damaging exercise that causes DOMS, with a gradual recovery in subsequent days. The magnitude and the recovery rate from strength loss seem to be related to the training history of the muscle group. For instance, when performing maximal eccentric contractions, upper limb muscles (less active) demonstrate greater loss of strength (50–70%) and slower recovery (60–90 days) when compared to lower limb muscles (locomotory muscles) (20–30% and 10–30 days, resp.) [37, 85]. However, only a few studies have investigated the effects of EIMD and DOMS on aerobic performance indexes (e.g., VO_2_max, lactate response to exercise, VO_2_ kinetics, and movement economy) [86]. These studies analysed the effects of EIMD on RE [10, 12] and VO_2_ kinetics during submaximal cycling exercise [86]. In this context, studies that investigated the effects of strength exercises (i.e., jumps, isoinertial and isokinetic eccentric exercises) on RE will be addressed (Table 1).

 Paschalis et al. [10] analysed the effects of eccentric exercises on indirect muscle damage markers (CK, DOMS, ROM, and isometric force) and RE in active individuals who were not engaged in strength training programs. The eccentric exercise protocol consisted of 120 (12 × 10) maximal voluntary contractions (MVC) at an angular velocity of 1.05 rad·s^−1^. Although indirect muscle damage markers were significantly altered in the subsequent days (24–72 h), the RE (assessed at 55 and 75% VO_2_max) was not modified. Similar data were obtained by Vassilis et al. [87], who analysed the effects of eccentric exercise (120 MVC at a 60°·s^−1^) on RE in recreational athletes with no previous experience in resistance training. The RE (assessed at 70% VO_2_max) was not changed 48 hours after the damaging bout. Therefore, EIMD induced by isokinetic eccentric contractions do not seem to interfere on RE measured at moderate intensities (55–75% VO_2_max).

 Using closed kinetic-chain exercises, Scott et al. [88] have also analysed the effects of EIMD on RE. The volunteers performed a series of lower extremity resistance exercises designed to induce DOMS. RE was analysed at 70% VO_2_max, 24–30 hours after the EIMD. Although the subjects demonstrated a higher rate of perceived exertion values, RE was maintained unaltered throughout the days after EIMD. In another study, Marcora and Bosio [9] did not find any alteration in RE (70% VO_2_max) after 100 drop jumps, although DOMS, CK, and knee extensors strength were significantly affected by EIMD. Therefore, the evidence suggests that muscle damage induced by both open and closed kinetic-chain exercises dose not alter RE at moderate intensities (55–75% VO_2_max).

 However, in a recent study, Burt et al. [12] presented conflicting data regarding the effect of EIMD on RE. In this study, indirect markers of muscle damage and RE were measured, 24–48 h after EIMD (10 sets of 10 squats at 80% body mass). Significant increases in all indirect markers of muscle damage, kinematic parameters (stride length and stride frequency), and oxygen uptake during submaximal running (~90% VO_2_max) were observed at 24–48 h following the initial bout of EIMD. Some authors [82, 89] have suggested that the changes in RE are associated with decrements in neuromuscular function (i.e., MVC) after EIMD. However, both the magnitude and the time course of the changes in muscular function (MVC) and RE can be different. Therefore, changes during submaximal exercise (i.e., RE) may not be strictly associated with neuromuscular function.

As a whole, these data suggest that the effects of muscle damage induced by strength exercise (i.e., jumps, isoinertial and isokinetic eccentric exercises) on RE are intensityddependent. During moderate exercises (55–75% VO_2_max), RE is not altered by EIMD. However, during high-intensity exercise (~90% VO_2_max), RE is impaired. During high intensity exercise, additional type II fibres, which are the most affected by EIMD, are recruited. Moreover, at these intensities, the VO_2_ either attains a delayed steady state (heavy domain) or continues to increase slowly (i.e., VO_2_ slow component (VO_2_SC)) reaching its maximal values at the end of exercise (severe domain) [90]. Although the physiological determinants of VO_2_SC remain poorly understood, some authors have proposed that an increased ATP and/or O_2_ cost of power production in fatigued fibres, rather than the additional recruitment of poorly efficient muscle fibres, is responsible for the VO_2_SC [91]. Therefore, the effects of strength exercise-induced muscle damage on RE seem to depend on the fibre recruitment pattern and/or on the mechanisms determining the VO_2_SC.

## 5. Downhill and Long-Distance Running, Muscle Damage, and Running Economy

 Adopting a more specific approach, some studies have investigated the influence of muscle damage induced by strenuous exercise (e.g., long-distance running) or downhill running on neuromuscular parameters, and RE. This aspect and the effect of some interventions on RE during the recovery period after EIMD are also addressed in this topic (Table 2).

As mentioned previously, muscle damage is usually induced by maximal and submaximal eccentric contractions, but it can also be observed when a high volume of eccentric/concentric contractions are performed, due to the eccentric contractions per se [95] or because of metabolite accumulation that may lead to stress and impairment of the muscle fibres [96]. Because a high number of concentric and, particularly, eccentric contractions are performed during long-distance running, the symptoms of muscle damage are usually observed immediately and a few days after the running bout. 

 In a study conducted by Millet et al. [97], changes in muscle function and muscle damage markers from 22 experienced marathon runners were collected and analysed after they had run an international extreme mountain ultramarathon. The race consisted of a 166 km marathon through mountainous terrain with the final destination set at 9500 m below the starting point. This predominately downhill configuration required a high number of eccentric contractions particularly for the knee extensors. Indirect muscle damage markers (strength, CK, LDH, and MB) were analysed before, immediately after, and 2, 5, 7, 9, and 16 days after the marathon. The authors found higher decreases in force production immediately after the ultramarathon, most likely because of the fatigue experienced during the race. However, some of the strength markers remained altered until 5 days after exercise, as usually occurs after muscle damaging activities. Blood markers also demonstrated the highest value immediately after the race, returning to baseline values 5 days later. The authors found that even though this type of activity can induce extreme muscle damage, after 16 days, all the alterations induced by muscle damage and/or fatigue had returned to normal. Considering that force production is intimately related to RE, these findings may indicate that an extremely damaging activity may induce high levels of force loss and decreases in RE. Force production is usually fully recovered 5 days after the damaging activity. However, RE may recover at a faster rate than force.

 To investigate factors that could influence RE, Kyrolainen et al. [89] subjected 7 experienced runners to a protocol simulating a marathon. RE and kinematic variables (stride length and frequency, mean contact time, external mechanical work and power, and angular displacements and velocities of the hip, knee, and ankle joints) were collected before, during (at the 1st, 13th, 26th, and 42nd kilometres), two hours after, and in the days (2, 4, and 6) after the marathon. Muscle damage markers (CK and SOR) were also collected after the exercise. The impairment of RE (i.e., higher oxygen consumption) was observed only at the end of the marathon (42nd kilometre and two hours afterward). CK and SOR were significantly increased immediately after the marathon and returned to baseline values only at the 6-day postexercise time point. These data may indicate that alterations in RE after marathon running may not be exclusively in the result of muscle damage but may be affected by other factors such as thermal stress. To better understand the time course of recovery of the various parameters of muscle function following marathon running, it is important to investigate other indirect EIMD markers, such as force and inflammatory response.

 Muscle damage induced by downhill running has also been widely studied in the last decades. This type of exercise has been proven to induce muscle damage even when performed for relatively short periods (e.g., 30 minutes) due to higher mechanical stress applied to the lower limb muscles during the contact with the ground phase [95]. Some studies have shown that downhill running can lead to muscle damage of the same magnitude as plyometric or maximal eccentric exercises [92, 98]. Considering that downhill running induces muscle damage, a series of studies have investigated its influence on neuromuscular and metabolic markers in animals [99] and humans [11, 93, 94, 98]. In animals, downhill running has been utilised to induce overtraining [100] as well as a training method to increase the number of sarcomeres [101]. In humans, this exercise model has been recently studied in attempt to understand its influence on specific running and aerobic parameters, such as RE [93, 98] and running kinematics.

 To the best of our knowledge, Hamill et al. [92] performed the first study investigating the influence of downhill running on RE. In this study, 10 recreational female runners underwent a 30 min downhill running bout (DRB) with −15% slope at 73.5% of maximal heart rate. Indirect markers of muscle damage (SOR and CK), RE (80% VO_2_max), and kinematic parameters were measured before and 2 and 5 days after the DRB. SOR and CK levels increased 2 days after the DRB, returning to baseline values 5 days after the exercise. Although kinematic parameters were modified, the DRB did not alter RE. The authors proposed that changes in kinematics might be due to increases in SOR, which compromises the range of motion, and thus alters the movement patterns. 

 In another model to investigate the influence of downhill running-induced muscle damage on RE, Braun and Dutto [93] conducted a study in which 9 endurance-trained subjects underwent a DRB (30 minutes at 70%  VO_2peak_ with a −10% slope). Assessments of SOR, RE (65%, 75% and 85% VO_2_max), and stride length were performed before and 48 hours after the DRB. SOR was increased and RE was impaired 48 hours after the DRB, suggesting that muscle damage might have increased the energy cost of running. The authors stated that the muscle damage decreased the range of motion and strength, thus compromising running kinematics, which is known to be related to RE.

 To better describe the time course of changes in RE, Chen et al. [94] subjected 10 soccer-trained volunteers to a downhill running protocol similar to that proposed by Braun and Dutto [93]. Muscle damage (MVC, SOR, CK and MB) and RE (65%, 75%,d and 85% VO_2_max) were assessed before and 1 hour and 1–5 days after the DRB. Alterations in muscle damage markers were consistent with those found in the literature, including increases in CK, MB, and SOR, with peak values attained 48 hours after the DRB. Strength loss was also maximal immediately after the DRB. All muscle damage markers returned to baseline values 5 days after the DRB. The magnitude of change was smaller, and the time course recovery was faster for RE (4–7% and 4 days, resp.) than for the indirect markers (i.e., isometric peak torque (IPT)) of muscle damage (7–21% and 4 days, resp.). The authors suggested that the alterations in running kinematics, the need to recruit more muscle fibres, the impairment in the stretch-shortening cycle, and the reduced levels of muscle glycogen might impair RE following a DRB.

 Because alterations in the muscular tissue due to EIMD have been shown to affect RE because of differences in muscle fibre recruitment and other neuromuscular properties, Chen et al. [11] assessed RE at 3 different intensities (70, 80, and 90% VO_2peak_) after a DRB. Muscle damage markers showed the expected alterations, peaking 2 days after DRB. The alteration in RE measured at 90%  VO_2peak_ was significantly higher than at 80%  VO_2peak_. No significant change in RE was found at 70%  VO_2peak_. Previous studies have indicated that fast-twitch motor units are progressively recruited with increased levels of exercise intensity [102]. Because several investigations have reported selective damage to type II muscle fibres after eccentric muscle actions in humans [40, 103], there appears to be a relationship between the motor unit recruitment pattern and impairment in RE. 

 Therefore, the effects of strength exercises and downhill running on RE seem to be different. While strength exercises seem to affect RE only during high intensity submaximal exercises (~90% VO_2_max), downhill running also increases the energetic cost during moderate exercises (>65% VO_2_max). It is important to note that during running exercise, the VO_2_SC is attenuated (heavy intensity) and/or nonexistent (moderate). Thus, the effect of strength exercises on RE seems to occur only at running intensities at which the VO_2_SC is present. Greater muscle mass and/or the magnitude or specificity of muscle damage induced by downhill running may partially explain these results.

A variety of interventions have been proposed to enhance recovery from EIMD, that is, to reduce the severity and duration of injury and SOR. It is a common belief that low-intensity training (i.e., active recovery) enhances the recovery process by accelerating the return to homeostasis after EIMD. To investigate whether submaximal running would influence the recovery from DRB, Chen et al. [98] analysed the effect of 30-minute daily running exercises performed at different intensities (40%, 50%, 60%, and 70%  VO_2peak_) by different groups on the recovery of muscle damage and RE. Muscle damage was induced by a DRB (30 minutes at 70%  VO_2peak_ with −10% slope). The authors found that the time-course recovery of muscle damage markers and RE was similar for all groups, regardless of whether submaximal running was performed. Thus, low-to-moderate-intensity running seems not to improve the recovery from muscle damage and/or RE impairment. 

 Performing a similar subsequent bout of eccentric exercise results in significantly less change in the markers of muscle damage. This phenomenon is known as the RBE [104]. In fact, Byrnes et al. [105] and Chen et al. [66] demonstrated that when a DRB was repeated 1–6 weeks after the first bout, the indirect markers of muscle damage (isometric peak torque, CK, SOR, and range of motion) were significantly reduced. Moreover, Chen et al. [106] verified that the RBE was also observed in RE and running kinematics parameters. In this study, 12 male subjects underwent the same downhill running protocol adopted by Chen et al. [11] except the interval allowed between bouts that was twice as long, to allow full recovery from the first bout. The authors found significant changes in all markers of muscle damage after both protocols. However, the RE, kinematics parameters, and SOR were less affected after the second DRB. Therefore, one bout of DRB might induce a protective effect, leading to reduced levels of SOR and blunted changes in RE and biomechanical parameters. 

 In another study, Burt et al. [12] subjected 9 subjects to repeated bouts of 100 squats and measured muscle damage markers (isometric peak torque, vertical jump height, CK, and SOR) and RE before, immediately after the bouts, and 1-2 days after the bouts. The bouts were separated by enough time to recover from the EIMD symptoms. All muscle damage and RE markers were significantly affected by the first bout. However, no alterations in some muscle damage markers and RE were observed after the second bout. Thus, a previous damaging activity leads to blunted or nonexistent alterations in muscle damage markers and RE.

## 6. Supplementation and Muscle Damage Recovery

 A series of recent studies has investigated the influence of different types of supplementation on recovery from and prevention of muscle damage. The main supplements that seem to protect against muscle damage are the flavonoids, which are known for their efficient anti-inflammatory and antioxidant properties. Studies investigating supplementation with flavonoid rich substances and their influence on muscle damage will be discussed.

 Howatson et al. [107] conducted a study in which muscle damage, inflammatory response, and oxidative stress were measured before, immediately after, and 24 and 48 hours after a marathon. The purpose of this study was to investigate whether a tart cherry juice supplement would affect recovery from muscle damage after marathon running in 20 recreational marathon runners, using a double-blind placebo intervention. Muscle damage markers determined in this study were CK, LDH, DOMS, and IPT. Other parameters (total antioxidant status, thiobarbituric acid reactive species,d and protein carbonyls) were measured to identify inflammatory response, and oxidative stress. Both groups (control versus supplemented) demonstrated similar decreases in IPT. However, IPT was higher for the supplementation group at all time points, showing faster recovery. Moreover, the authors found that the supplementation enhanced the anti-inflammatory response as well as reduced the oxidative stress. 

 Kuehl et al. [108] investigated the effects of tart cherry juice supplementation on SOR immediately after a long-distance running (~26 km) bout. The study was performed in a randomised, double-blind placebo fashion in 54 experienced runners. The subjects were separated in two groups: placebo and tart cherry supplement. Both groups started ingesting their supplements 7 days prior to the running bout. The increase in SOR was significantly greater for the placebo group when compared to the tart cherry group. These findings are similar to those of Howatson et al. [107] and indicate that the anti-inflammatory and antioxidant properties of the tart cherry supplement might reduce SOR after EIMD.

 Supplements containing flavonoid compounds have been shown to confer a protective effect against muscle damage either by attenuating a vast number of markers or by accelerating their recovery after the EIMD. This type of protection has been hypothesised to be due to the anti-inflammatory and antioxidant properties present in these types of compounds [107]. A number of studies have shown a direct relationship between flavonoid supplementation and protection against muscle damage. Because muscle damage may affect RE, it would be interesting to analyse if this type of supplementation can protect against RE impairment after EIMD.

## 7. Conclusion

 Despite the systematic implications of RE on aerobic running performance, only a few experiments have specifically studied the response of this index after EIMD. Recent studies have analysed RE after strength exercises (i.e., jumps, isoinertial and isokinetic eccentric exercises) and downhill running. These studies have found that the magnitude of reduction in muscle function (MVC) after EIMD is greater than the RE and kinematic parameters. Moreover, the time course for the changes in muscle function, RE, and kinematic parameters are not similar. As a whole, these data suggest that the putative mechanisms underlying muscle function and RE during the recovery from EIMD are not completely shared. The effects of muscle damage on RE seem to depend of the interaction between the type of eccentric exercise and the intensity at which the RE is measured. Strength exercises seem to modify RE preferentially during high-intensity exercise (~90% VO_2_max). However, the effects of downhill running can also be observed at moderate intensities (>65% VO_2_max). Finally, a single session of strength exercise or downhill running attenuates changes in indirect markers of muscle damage and blunts changes in RE. 

## Figures and Tables

**Table 1:** Comparison of the effects of the resistance exercise on running economy.

Study	Subjects	EIMD	Muscle damage	VO_2_max (%)	RE (%)
Paschalis et al. [10]	10 healthy males	120 eccentric actions	↑ CK, ↑ DOMS, and ↓ ROM, and ↓ strength	55 and 75	√
Burt et al. [12]	9 healthy men	100 squats at 80% body mass	√ CK, ↑ DOMS, and ↓ strength	90	↓ 4-5
Vassilis et al. [87]	24 young healthy men	120 eccentric actions	↑ CK, ↑ DOMS, ↓ strength	70	√
Scott et al. [88]	8 active men and 8 active women	3-4 × 10 repetitions of squat, lunges, step up and step down, and stiff-legged deadlift	↑ DOMS	70	√

EIMD: exercise-induced muscle damage; %VO_2_max: exercise intensity at which running economy was measured; RE: running economy; CK: creatine kinase; DOMS: delayed onset muscle soreness; ROM: range of motion; ↓ indicates decrease; √ indicates no change; ↑ indicates increase.

**Table 2:** Comparison of the effects of the downhill running on running economy.

Study	Subjects	EIMD	Muscle damage	VO_2_max (%)	RE (%)
Chen et al. [11]	50 male students	30 min DHR at −15%	↑ CK, ↑ DOMS, ↓ strength, and ↑ LDH	70, 80, and 90	↓ 5
Hamill et al. [92]	10 recreational female runners	30 min DHR at −15%	↑ CK, ↑ DOMS	80	√
Braun and Dutto [93]	9 endurance trained men	30 min DHR at −10%	↑ DOMS	65, 75, and 85	↓ 3
Chen et al. [94]	10 soccer trained men	30 min DHR at −15%	↑ CK, ↑ DOMS, ↓ strength, and ↑ MB	65, 75, and 85	↓ 4–7

EIMD: exercise-induced muscle damage; DHR: downhill running; %VO_2_max: exercise intensity at which running economy was measured; RE: running economy; CK: creatine kinase; DOMS: delayed onset muscle soreness; MB: myoglobin; LDH: lactate dehydrogenase; ↓ indicates decrease; √ indicates no change; ↑ indicates increase.
